# Diving into discomfort: orofacial pain dynamic—A systematic review

**DOI:** 10.3389/fpubh.2025.1553541

**Published:** 2025-05-09

**Authors:** Lubna A. Alolaiwi, Fawziah A. Alzahrani, Sulaiman A. AlShammari

**Affiliations:** ^1^Department of Dental Public Health, College of Medicine and Dentistry, Riyadh Elm University, Riyadh, Saudi Arabia; ^2^Department of Family and Community Medicine, College of Medicine, King Saud University, Riyadh, Saudi Arabia

**Keywords:** orofacial pain, diving, barotrauma, barodontalgia, periodontal issues, temporomandibular joint, hyperbaric dentistry, systematic review

## Abstract

**Introduction:**

Scuba diving is a popular recreational activity. However, it carries inherent risks, including exposure to hyperbaric environments, which can exacerbate medical conditions, such as dental barotrauma, barodontalgia, temporomandibular disorders, and periodontal issues. Understanding the prevalence of orofacial pain among divers is critical for improving diver safety and health. Thus, this study aimed to investigate the occurrence and contributing factors of orofacial pain in various diver populations to inform targeted preventive strategies.

**Methods:**

A systematic literature search was conducted across PubMed, Saudi Digital Library, and Google Scholar, and articles that studied orofacial pain among divers were selected. The Joanna Briggs Institute was used to assess the risk of bias. Due to insufficient statistical data (e.g., confidence intervals, standard errors) and extremely high heterogeneity (*I*^2^ values of 97.23% for barodontalgia and 98.03% for dental barotrauma), a meta-analysis was deemed inappropriate, a synthesis without meta-analysis was conducted to assess the prevalence of various types of orofacial pain across diverse diver populations.

**Results:**

This review included seven studies that examined orofacial pain prevalence and some risk factors (e.g., diving depth, frequency, occupational duration, and oral health condition) among military, occupational, and recreational divers. Barodontalgia had the highest prevalence rate among the four types of orofacial pain studied in this review, ranging from 10.8% to 56.1%, whereas periodontal issues showed the lowest rate, ranging from 2.8 to 6.6%, and were the least frequently studied type of pain. Military divers were most affected by all types of pain.

**Conclusion:**

This study underscores the necessity of tailored preventive strategies for divers, particularly military and leisure divers. These strategies should focus on dental care, ergonomic interventions, oral hygiene education, equipment fitting, and clenching management.

## 1 Introduction

Scuba diving is a popular recreational activity worldwide, with the Professional Association of Diving Instructors issuing ~29 million certifications in 2021 (1). While diving is considered safe with appropriate training and certification, it still involves inherent health risks. Divers are exposed to hyperbaric environments, such as atmospheres with increased pressure, which can significantly affect the body and exacerbate various medical conditions (2). Among these, orofacial issues, such as dental barotrauma, barodontalgia, and temporomandibular joint (TMJ) dysfunction (temporomandibular disorders [TMDs]), are increasingly recognized as significant challenges for divers, leading to pain, discomfort, and potential diving complications (3).

Changes in atmospheric pressure can influence gas expansion and compression within the dental structures, contributing to conditions, such as barodontalgia and dental barotrauma. Damage to teeth and dental reconstruction, known as dental barotrauma, can occur with or without pain when the surrounding pressure varies (4). Barodontalgia is an intraoral pain that occurs in an otherwise asymptomatic oral cavity and is triggered by a change in barometric pressure. Barodontalgia is often a symptom of a preexisting subclinical oral disease rather than a standalone condition. The most common dental diseases can be the cause of barodontalgia (4–6). Temporomandibular disorders (TMDs) may be triggered by the features of the divers' equipment in their oral cavities to breathe during immersion (7). These patients exhibit traits associated with abnormalities in the masticatory muscles, oral tissues, and TMJ. “Diver's mouth syndrome” is the term used to describe all these concerns (8).

This systematic review aimed to answer the following research question: What is the prevalence of orofacial pain among divers exposed to atmospheric pressure changes during diving, and what are the associated risk factors? Understanding the prevalence of orofacial pain among divers and its causes is similarly necessary for crafting better prevention efforts and minimizing diving hazards. Discussing variables of diving depth, diving frequency, and whether dives were for labor, this review aims to add information that will help improve diver safety and overall health.

## 2 Methods

This systematic review adhered to the Preferred Reporting Items for Systematic Reviews and Meta-Analyses (PRISMA) checklist (9) and Synthesis without Meta-Analyses guidelines (10).

### 2.1 Protocol and registration

While registration on PROSPERO or a similar registry is a best practice to support transparency and reproducibility, the process inadvertently was bypassed in the preliminary stages of the review process. However, we strictly adhered to the PRISMA and SWiM guidelines and documented every methodological decision, including search strategy, inclusion/exclusion, and data extraction, to facilitate rigor and transparency.

### 2.2 Population, exposure, and outcome framework

This systematic review focused on military, recreational, and occupational divers as the primary populations of interest. The exposure examined was the atmospheric pressure changes experienced during diving. The outcomes of interest were the prevalence and types of orofacial pain, such as barodontalgia, dental barotrauma, TMDs, and periodontal issues, reported among divers. As this review aimed to describe the prevalence and risk factors of orofacial pain in divers, no formal comparator group was included.

### 2.3 Eligibility criteria

Studies were eligible if they examined professional or recreational divers exposed to atmospheric pressure changes and the development of dental and orofacial conditions. Eligible studies were required to explore the relationship between pressure changes and the onset of dental or orofacial pain, including outcomes, such as the prevalence of dental barotrauma, barodontalgia, TMDs, and periodontal issues. Only quantitative studies that involved human participants were included. Studies published in English since 2020 were considered to ensure that the review reflected the most up-to-date knowledge. Laboratory or non-human studies, narrative reviews, studies published in non-peer-reviewed journals, and studies without empirical data were excluded.

### 2.4 Search strategy and selection

In October 2024, a comprehensive search strategy was applied using the Saudi Digital Library (SDL), PubMed, and Google Scholar as the three main databases. Since SDL offers access to several indexed databases such as Medline, Springer, ProQuest, Access Medicine, and ACM Digital Library, its utilization was especially important. This helped to capture a wide selection of peer-reviewed literature despite direct access limitations to specific databases (e.g., Scopus, Embase). Utilizing SDL's robust resources together with PubMed and Google Scholar, we aimed to limit potential gaps in study retrieval, as well as achieve a solid evidence base. We identified the search terms and combined them with the corresponding Boolean operators. The search sequence that was submitted was as follows: ((“dived” OR “dives” OR “diving” OR “Naval” OR “Scuba” OR “atmospheric pressure changes”) AND (“facial pain” OR “dental pain” OR “orofacial pain” OR “barotrauma” OR “barodontalgia” OR “temporomandibular joint dysfunction” OR “hyperbaric dentistry” OR “gum pain” OR “tooth fracture”)). The references cited in the articles were reviewed to identify additional relevant materials. Citations were imported into the EndNote 21 software (11), and duplicates were eliminated. A web-based software for systematic reviews was used to screen and assess identified studies (12). The screening was completed in two phases: title/abstract and full text. During all the rounds of the review process, two reviewers (LA and FA) independently evaluated the electronic search titles and abstracts and retrieved all articles. Disputes were settled via dialogue or consultation with a third author (SA). A flow diagram illustrating the research selection process was finalized using the PRISMA checklist (9, 13).

### 2.5 Quality assessment

Two reviewers (LA and FA) independently conducted quality evaluation. The Joanna Briggs Institute (JBI) (14) criteria were used. The tool consisted of eight components, with each item scored as “yes,” “no,” “unclear,” or “not applicable.” The study received one point for every affirmative (“yes”) response. Studies were not excluded depending on JBI scores per accepted criteria (15); rather, the findings regarding the methodological quality of included studies were reported for transparency. This approach ensures that all relevant studies are considered while allowing readers to interpret findings within the context of study quality.

### 2.6 Data collection

Data were collected on study characteristics (e.g., investigator, year, location, sample size), participant demographics (e.g., mean age and sex), diving variables, and risk factors (e.g., depth, frequency, occupational duration, gas type, and orofacial pain types). Two reviewers (LA and FA) independently extracted the data, and a third reviewer (SA) resolved any discrepancies. The results were compiled into a table using Microsoft Excel (16).

### 2.7 Data synthesis and heterogeneity assessment

In this review, a meta-analysis could not be performed because many studies did not report the statistical data required, such as confidence intervals or standard errors. To assess the feasibility of a meta-analysis, a heterogeneity assessment was conducted for the two most frequently reported outcomes: barodontalgia and dental barotrauma. The results revealed extremely high heterogeneity, with *I*^2^ values of 97.23% for barodontalgia and 98.03% for dental barotrauma. This indicates that the differences in study results were not random; rather, they reflected real differences in study populations, methodologies, or other factors. Given this level of heterogeneity, even if the necessary data had been available, a meta-analysis might have produced misleading results.

Instead, the Synthesis Without Meta-Analysis (SWiM) approach was used to synthesize findings. Studies were first grouped by type of orofacial pain (e.g., barodontalgia, TMJ pain, dental barotrauma, periodontal) and subsequently stratified according to diver type (military, occupational, recreational) to identify differences in risk factors and prevalence. The results were synthesized narratively, describing patterns, trends, and salient risk factors. Summary tables and figures were used to report outcomes and allow for easier comprehension. Descriptive synthesis methods allowed data from various studies to be included based on high-quality studies that had the most significance for the question under consideration, as assessed using the JBI quality appraisal tools. Study quality and consistency of findings were considered when qualitatively appraising the quality of evidence.

## 3 Results

**Figure 1:**
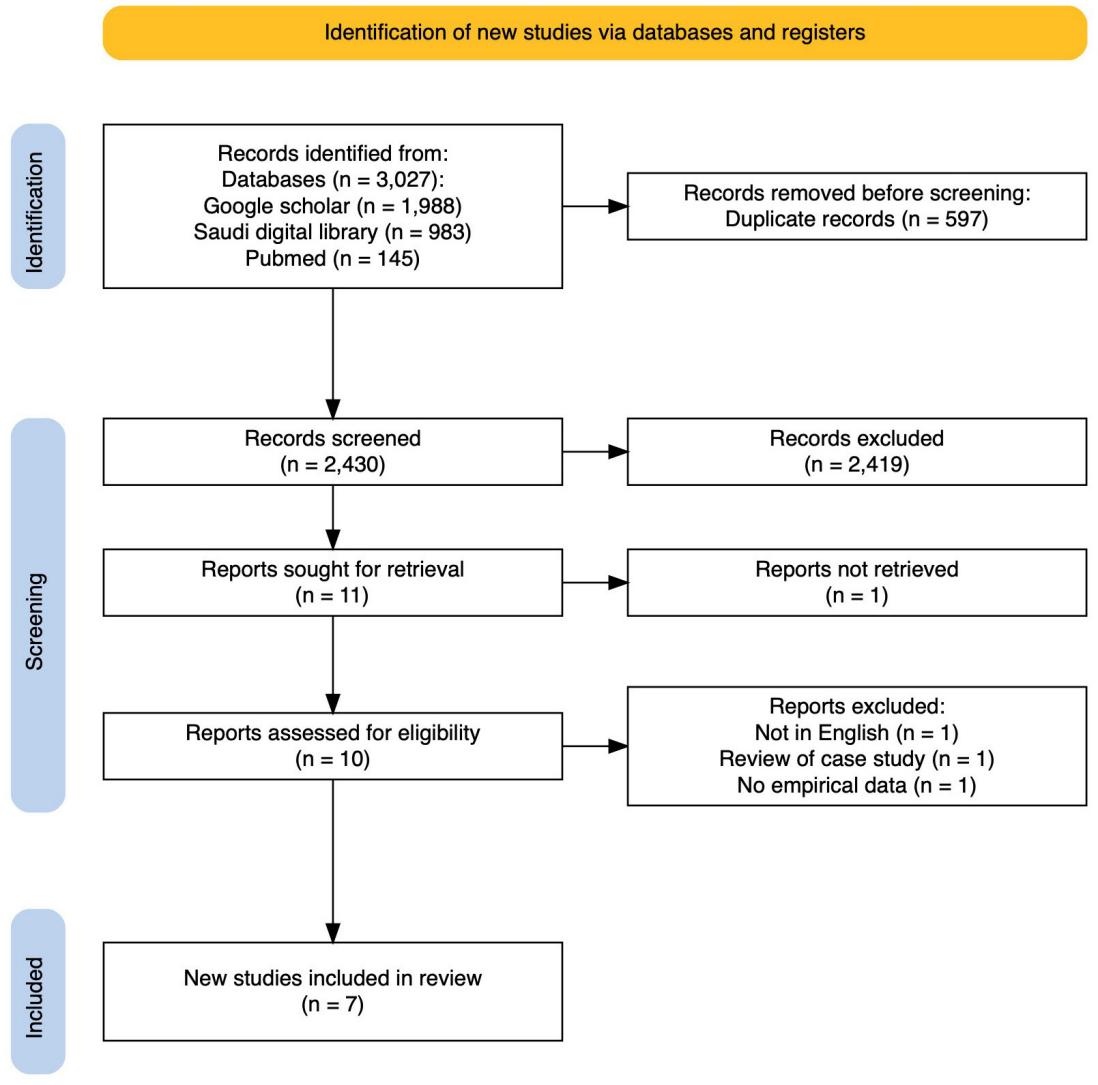
Preferred reporting items for systematic reviews and meta-analyses flowchart summarizes the search screening and selection of the reviewed articles.

The initial search identified 3,027 studies. After removing duplicates (*n* = 597), 2,430 records remained; after the second elimination round, based on a review of study titles and abstracts, we excluded 2,420 records based on our predefined eligibility criteria: studies not published in English, non-human or laboratory studies, narrative reviews or non-peer-reviewed publications, and studies unrelated to dental/orofacial conditions in divers. Ten studies met the eligibility criteria, and full texts were thoroughly examined. Three studies were excluded from the full-text stage because one (17) only had an abstract in English, and the full text was unavailable in English, the other was a review of two case studies not evident in the abstract (18), and the third did not provide empirical data (19). Finally, seven studies were included in this review. The PRISMA flowchart illustrates the screening and selection processes, as shown in Figure 1.

### 3.1 Characteristics of the included studies

All the studies included in this review were cross-sectional. Tables 1A, B summarizes the characteristics of the included studies. All studies were published between 2020 and 2023 and conducted in various countries; some were from Europe, others from Asia, and two were conducted in Arab countries. The sample sizes varied, with most studies comprising ~200 participants. The male participants outnumbered the female participants. The participants represented diverse groups, including military, occupational, civilian, and recreational divers. The ages of the participants ranged widely from 18 to 79 years, with the mean ages reported between 22.1 and 48 years. The JBI tool for cross-sectional studies was used to assess the quality of the included studies, with scores ranging from 5 to 8/8. A detailed summary of these assessments is presented in Table 2.

**Table 1:** Characteristics of included studies.

**A**								
**Study identification**	**Country**	**Study design**	**Participants**	**Sample size**	**Age (mean** ±**SD/95% CI/range)**	**Sex distribution**	**Diving-related variables**	**Orofacial pain types**
Alwohaibi et al. (20)	Saudi Arabia	Cross-sectional	Military divers	216	Range: 25–50 (96.9%)	100% male	Military diving, diving depth, frequency	Barodontalgia, TMJ, dental barotrauma
Onose et al. (27)	Japan	Cross-sectional	Occupational divers	242	Range: 20–79 years	100% male	Occupational diving, diving frequency, duration	Barodontalgia, TMJ, dental barotrauma, periodontal issues
Shshtari et al. (26)	Kuwait	Cross-sectional	Instructors, dive masters, advanced open water divers, rescue divers, and open water divers	150	Mean: 32.3 ± 7.2	69.3% male, 30.7% female	Scuba diving, diving frequency	Barodontalgia, periodontal issues
Moyaux et al. (3)	France	Cross-sectional	Civilian scuba divers	1,015	Range: 18–34 years	65.4% male, 34.6% female	Scuba diving, diving level, diving habits	Barodontalgia, TMJ diver's mouth syndrome, dental barotrauma, periodontal issues
Tsur et al. (32)	Israel	Retrospective study	Military divers and nondivers	1,036 divers out of 6398 soldiers	Mean: 22.1	100% male	Military divers, dental health, age	Dental barotrauma, TMJ
Gougeon et al. (28)	France	Cross-sectional	Civilian divers	684	Mean: 48 ± 12	65.4% male, 34.6% female	Scuba diving, diving level, age	Barodontalgia, dental barotrauma
Sumen et al. (29)	Turkey	Survey study	Divers (instructors, recreational, commercial)	287	Mean: 38.96 ± 12.64	79.1% male, 20.9% female	Scuba diving, diving frequency, diving depth	Barodontalgia, TMJ diver's mouth syndrome, periodontal issues, dental barotrauma
**B**								
**Study identification**	**Pain prevalence**			**Risk factors**	**Pain measurement**	**Statistical analysis used (** ***p*** **-value)**	**Outcome**	**Study quality**
Alwohaibi et al. (20)	Barodontalgia (“tooth pain”) (56.1%), dental barotrauma “dental injury” (52.3%), TMJ pain, “TMJ pain or clicking” (13.9%)			Frequency of diving, depth, atmospheric pressure during diving, age	Self-reported questionnaires	Descriptive, Kolmogorov–Smirnov, Kruskal–Wallis test (*p < * 0.05)	Pain levels were significantly higher among divers diving >10 times per year, at depths >20 m, and with atmospheric pressure >1.5 bar	6 out of 8
Onose et al. (27)	Barodontalgia (“tooth pain”) (17.8%), TMJ pain (15.7%), dental barotrauma (“detachment of inlays or crowns”) (12.0%), periodontal issues (“painful gums”) (6.6%)			Frequency of diving, dental health, age, diving duration	Self-reported questionnaire	Descriptive, Fisher's exact test (*p < * 0.05), Spearman's correlation coefficient, logistic regression	Preventive dental visits reduce symptoms	7 out of 8
Shshtari et al. (26)	Barodontalgia: “toothache” (16.2%), “tooth sensitivity” (3.0%), periodontal issues (“shooting pain”) (6.3%).			Frequency of diving, age, diving experience, dental consultation	Self-reported questionnaire	Descriptive, chi-squared, Kruskal–Wallis test (*p < * 0.05)	Toothache more common in younger divers	7 out of 8
Moyaux et al. (3)	TMJ pain (“mouth syndrome”) (13.4%), barodontalgia (“tooth pain”) (10.8%), dental barotrauma: “barotrauma” (3.7%), “dental fracture caused by a shock” (1.9%), periodontal issues (“gum pain”) (2.8%)			Sex, “age,” “diving level,” diving frequency, knowledge, and prevention	Self-reported questionnaire	Descriptive, chi-squared, Fisher's exact test (*p < * 0.05), logistic regression	Prevention through dental checkups	7 out of 8
Tsur et al. (32)	Dental barotrauma (“faulty dental restorations”) (9.3%), TMJ pain (“disc dislocation without reduction”) (0.4%), TMJ pain (“disc dislocation with reduction”) (0.9%)			Oral health, temporomandibular disorders (TMDs), diving experience, age	Dental files, retrospective analysis	Descriptive, bivariate analysis, logistic regression (*p < * 0.05)	Higher risk of oral pathoses in military divers	8 out of 8
Gougeon et al. (28)	Barodontalgia (“tooth pain”) (18.7%), dental barotrauma: “loss or fracture of a dental filling” (4.2%), “dislodgement of a crown or bridge” (2.3%), “tooth fracture” (2.0%)			Diving experience, sex, age, diving depth, dental restorations, diving qualification, frequency of diving	Self-reported questionnaire	Chi-squared test, Fisher's exact test, and Mann- Whitney test (*p < * 0.05).	Need for regular dental checkups	7 out of 8
Sumen et al. (29)	TMJ pain (“joint sounds in daily life”) (35%), “jaw and masticatory muscle pain in the morning” (28.8%), “limitation of mouth opening in daily life” (21.1%), barodontalgia (“dental pain”) (22.6%), dental barotrauma (“restoration fracture”) (17.5%), “tooth fracture” (2.8%), “loss of unremovable prosthesis” (2.3%)			Diving experience, diving depth, diving duration, dental hhealth behaviors, sex, age, smoking habits, alcohol consumption	Retrospective self-reported questionnaire	Mann Whitney U test, Descriptive, chi-squared test, Fisher Freeman Halton (*p < * 0.05).	Exacerbation of TMJ pain after diving	5 out of 8

**Table 2:** Risk-of-bias assessment for included studies.

**Study**	**Q1: defined criteria**	**Q2: subjects and setting**	**Q3: valid exposure**	**Q4: standard criteria**	**Q5: confounders**	**Q6: strategy for confounders**	**Q7: valid outcomes**	**Q8: statistical analysis**	**Total score**
Alwohaibi et al. (20)	Yes	Yes	Yes	Yes	No	No	Yes	Yes	6
Onose et al. (27)	Yes	Yes	UC	Yes	Yes	Yes	Yes	Yes	7
Shshtari et al. (26)	Yes	Yes	UC	Yes	Yes	Yes	Yes	Yes	7
Moyaux et al. (3)	Yes	Yes	UC	Yes	Yes	Yes	Yes	Yes	7
Tsur et al. (32)	Yes	Yes	Yes	Yes	Yes	Yes	Yes	Yes	8
Gougeon et al. (28)	Yes	Yes	UC	Yes	Yes	Yes	Yes	Yes	7
Sumen et al. (29)	Yes	Yes	UC	Yes	No	No	Yes	Yes	5

UC, unclear.

The included studies used various tools to measure orofacial pain in divers. The study (20) used a questionnaire developed according to military diving medical standards (21–25) to assess dental and TMJ symptoms. Another study (26) adapted questions from Jagger et al. (7) and included additional customized items designed by an experienced diver. However, Onose et al. (27) used a comprehensive questionnaire to evaluate dental and TMJ symptoms issues, specifically targeting occupational divers. A French study (3) developed and piloted an online questionnaire to collect binary data on orofacial issues and dental fractures. Similarly, a second French study (28) created and tested a questionnaire focusing on barodontalgia and dental trauma, refining it for clarity and alignment with the study objectives. However, Sumen et al. (29) used a retrospective questionnaire to evaluate diving-related orofacial injuries, which is grounded in the existing literature (18, 20, 30, 31). Tsur et al. (32) relied on digital dental records to analyze orofacial symptoms in navy personnel. Finally, the retrospective case study by Sumen et al. (29) used descriptive data but did not rely on validated tools.

### 3.2 Grouping of studies for synthesis

**Figure 2:**
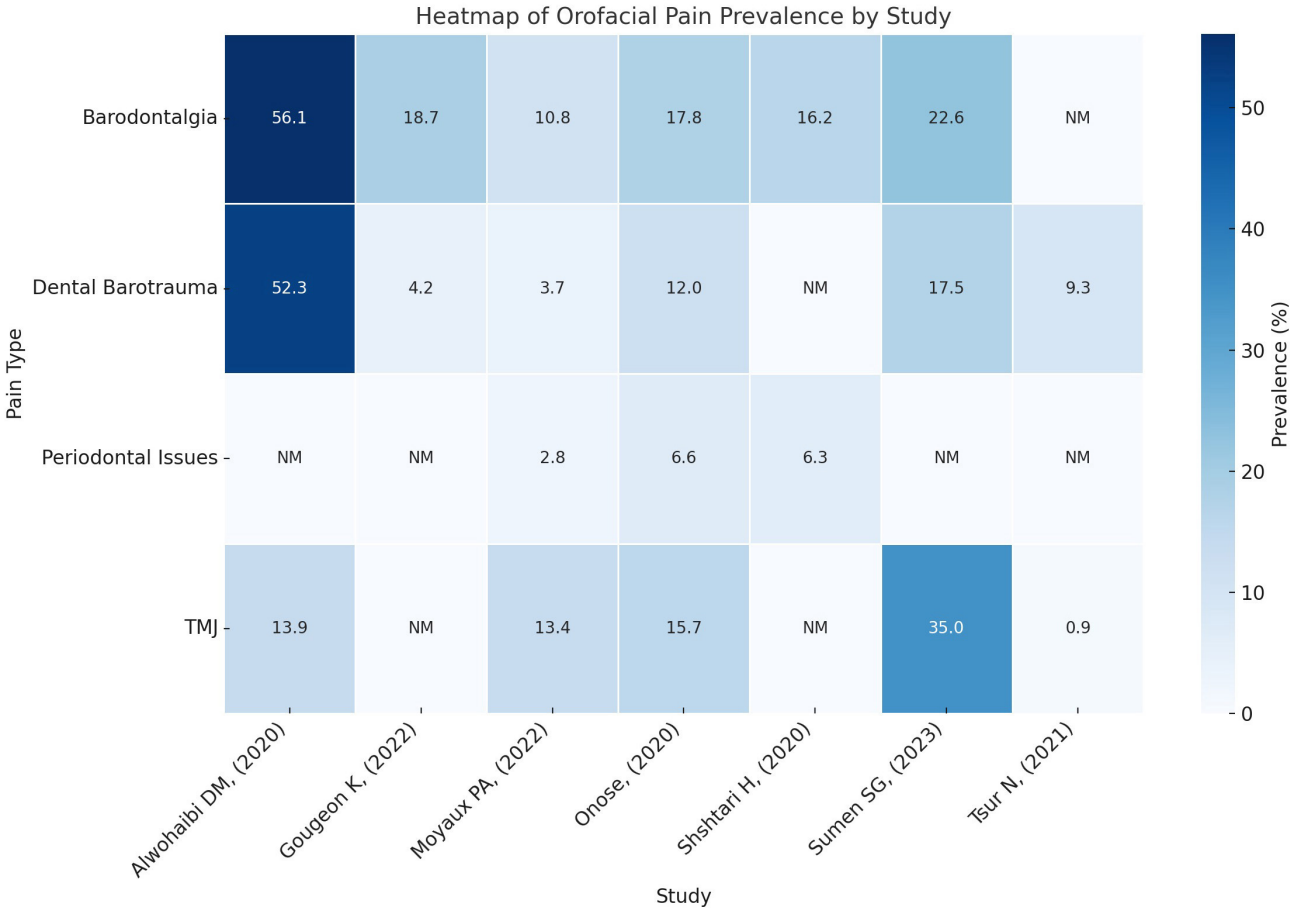
Heatmap representation of orofacial pain prevalence grouped based on pain type and study. Rows correspond to pain types, and columns represent studies arranged chronologically. The intensity of the color reflects the prevalence rate, with darker shades indicating higher values. Missing data are annotated as “NM” (not mentioned).

Studies were grouped according to the type of orofacial pain reported to facilitate the comparison of prevalence rates and associated risk factors. Additionally, participant characteristics and diving-related variables were considered to explore their influence on the prevalence of orofacial pain. The groupings and prevalence rates are shown in Figure 2.

### 3.3 Prevalence of orofacial pain

#### 3.3.1 Barodontalgia: “tooth pain”

Barodontalgia, commonly referred to as tooth pain, was the most frequently reported orofacial issue across five studies, with prevalence rates ranging from 10.8% to 56.1%. Alwohaibi et al. (20) reported the highest prevalence (56.1%) among military divers, with higher pain levels in those who dived 10–50 times per year and >50 times per year. Divers who dived at depths of 20–50 m and >50 m and those exposed to higher atmospheric pressures (1.5–2 bar, 2.1–3 bar, and >3 bar) experienced more severe pain. Onose et al. (27) found that 17.8% of occupational divers reported tooth pain, with frequent diving (four or more times per week) being significant risk factors, while dental consultations for prevention within the past year were a significant protective factor. In this study, older divers were more likely to experience tooth pain than younger divers. Shshtari et al. (26) demonstrated that 16.2% of divers, including instructors, dive masters, and rescue divers, experienced tooth pain. Frequent divers (≥4 dives a week) and those with longer dive durations (≥300 min) were at a higher risk, with younger divers (≤ 30 years) particularly affected. Moyaux et al. (3) found that 10.8% of civilian divers reported tooth pain, with older divers (age ≥65 years) showing a higher prevalence. Divers with higher diving certifications (such as 4th-level certification) also had higher rates of barodontalgia. Finally, Gougeon et al. (28) found that 18.7% of civilian divers reported tooth pain, with shallow-water dives (≤ 20 m) being associated with increased pain in 73.4% of cases. Dental restorations, including fillings, were a significant risk factor for barodontalgia because the air trapped under the restorations expands and contracts with pressure changes. Overall, studies have highlighted that frequent diving, greater depth, and existing dental restorations are significant risk factors for tooth pain, and older and more experienced divers are more likely to experience this condition.

#### 3.3.2 TMJ pain

TMJ pain was reported in five studies, with a prevalence ranging from 0.4% to 35%. Alwohaibi et al. (20) found that 13.9% of military divers reported TMJ pain, with a higher prevalence among divers aged 25–45 years and those diving at greater depths under higher atmospheric pressures. Onose et al. (27) observed 15.7% of occupational divers with TMJ pain, noting that frequent diving and longer dive durations (≥300 min) were key risk factors. Older divers (age ≥45 years) were also more affected by jaw strain from extended dives than younger divers. In the study of Moyaux et al. (3), 13.4% of civilian divers experienced mouth syndrome, with women reporting higher rates of jaw pain (18.6%) than men and younger divers (age 18–34 years) being more prone to this syndrome than older divers. Diversity with lower certification also showed a higher prevalence. Tsur et al. (32) reported that 0.4% of military divers had disc dislocation without reduction and 0.9% had disc dislocation with reduction, primarily associated with diving experience and the physical stress of diving equipment. Older divers and those with more diving experience were at greater risk than their counterparts. Sumen et al. (29) found that 35% of divers reported joint sounds in daily life, with female divers being significantly more affected than male divers. Diving experience, depth, and frequency contributed to TMJ, jaw, and muscle pain (28.8%) and limitation of mouth opening (21.1%). Longer dive durations and tight equipment were common factors that led to jaw strain and discomfort. Overall, TMJ pain was common, especially among more experienced divers, female divers, and divers diving at greater depths or for longer durations. Repetitive jaw strain and tight diving equipment were significant risk factors.

#### 3.3.3 Dental barotrauma (restoration fracture, tooth fracture, loss of prosthesis)

Dental barotrauma was reported in six studies, with prevalence rates ranging from 2.3% to 52.3%. 52.3% of military divers reported dental injuries, with a higher frequency of diving (10–50 times per year and >50 times per year) contributing to higher pain levels (20). Deeper dives (20–50 m and >50 m) and higher atmospheric pressures (1.5–3 bar and >3 bar) were also significant risk factors for dental injuries. Onose et al. (27) reported that in 12.0% of occupational divers, dental injuries were caused by the precise detachment of inlays or crowns. Diving duration (≥300 min per day) and frequent diving for work-related reasons were key risk factors. Regular dental consultation was recommended to reduce the incidence of such injuries. Moyaux et al. (3) reported that 3.7% of civilian divers experienced dental barotrauma, with restoration fractures being the most common. Older divers (particularly the ≥65-year age group) were more prone to dental barotrauma than younger divers due to wear on their dental restorations. More experienced divers (e.g., those with 4th-level certifications) also reported higher rates of dental injuries. According to Tsur et al. (32), 9.3% of military divers reported dental injuries, particularly faulty dental restorations. Oral health and the condition of dental restorations were key risk factors, with divers who had previous dental work being more vulnerable to barotrauma due to pressure changes affecting fillings and crowns than their counterparts. Gougeon et al. (28) found that 4.2% of civilian divers reported loss or fracture of dental fillings. Experienced divers, especially those with higher-level certifications, were at an increased risk owing to the cumulative effect of pressure changes over time. Dental restorations, particularly composite fillings, were more likely to fail under diving pressure. Older divers were also more susceptible to dental fractures due to natural wear and tooth tears on their teeth than younger divers. Finally, Sumen et al. (29) reported that 17.5% of divers experienced restoration fractures, with diving depth (>30 m), diving experience, and the condition of dental restorations being the key contributing factors. Similarly, 2.8% of the divers experienced tooth fractures, with deeper dives and existing dental work being significant risk factors. Of the divers, 2.3% reported a loss of unremovable prostheses, with diving experience and prosthesis quality being the key contributors to this issue. In summary, dental barotrauma is a significant concern for divers, with dental injuries ranging from tooth fractures to the loss of dental restorations. Risk factors include diving depth, frequency of diving, diving experience, and condition of dental restorations, with older divers and those with previous dental experience being particularly vulnerable to these issues.

#### 3.3.4 Periodontal issues: “gum pain”

Periodontal issues, particularly gum pain, were less frequently reported across the included studies. Only three studies documented gum pain symptoms, with prevalence rates ranging from 2.8% to 6.6%. According to Onose et al. (27), 6.6% of occupational divers reported painful gums. Older divers (age ≥45 years), those who dived more frequently, and those with longer diving durations were more likely to experience periodontal pain than their counterparts. Frequent diving and prolonged exposure to pressure changes during dives were significant risk factors for the exacerbation of gum issues. Similarly, Shshtari et al. (26) found that 6.3% of divers reported shooting pain in their gums. Again, older divers (age ≥45 years) were more susceptible than younger divers, with frequent diving and longer dives (≥300 min) contributing to increased periodontal pain. Prolonged pressure changes associated with diving were noted as exacerbating factors. According to Moyaux et al. (3), 2.8% of civilian divers experienced gum pain, with older divers (especially those aged ≥45 years), experienced divers, and female divers being more likely to report this issue than their counterparts. Prolonged exposure to pressure changes and previous diving experience increased the risk of gum pain, highlighting the importance of oral health maintenance for divers. Overall, periodontal issues in divers are associated with age, frequency and duration of diving, and diving experience, with older divers and those with more diving experience being more vulnerable than their counterparts. Exposure to pressure changes during diving likely contributes to gum discomfort and other related symptoms.

### 3.4 Risk factors

**Figure 3:**
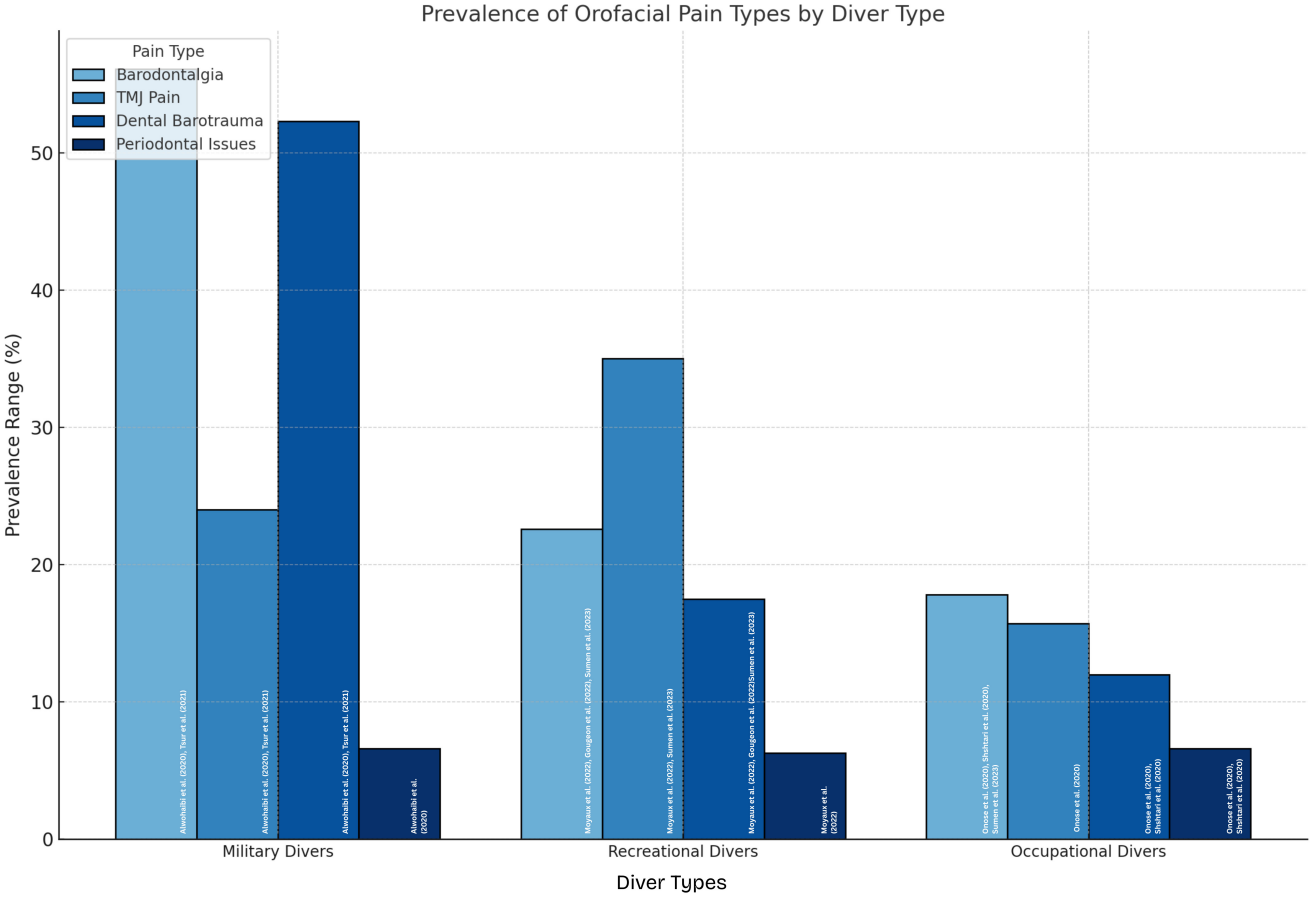
Prevalence of orofacial pain type among different diver types.

The risk factors contributing to orofacial pain in diverse species varied across studies and were strongly associated with the type of pain experienced. Barodontalgia was associated with untreated dental conditions, frequent deep dives, and poorly sealed restorations. TMJ pain was influenced by jaw clenching, repetitive strain, and equipment tightness. Dental barotrauma was significantly associated with diving depth, frequency, and existing dental restorations, whereas periodontal issues were associated with poor oral hygiene and prolonged exposure to pressure changes. These findings are summarized in Table 3, and a graphical representation of the diver types and their associated orofacial pain prevalence is shown in Figure 3.

**Table 3:** Risk factors based on pain type.

**Pain type**	**Prevalence range (%)**	**Risk factors**	**Associated studies**
Barodontalgia	10.8–56.1	Frequent diving, greater depths (>20 m), higher atmospheric pressures (>1.5 bar), untreated dental caries, dental restorations	Alwohaibi et al., Onose et al., Shshtari et al., Moyaux et al., Gougeon et al.
TMJ pain	0.4–35	Jaw clenching, tight equipment, frequent long dives (>4 dives/week, >300 min), female divers, older divers	Alwohaibi et al., Onose et al., Moyaux et al., Tsur et al., Sumen et al.
Dental barotrauma	2.3–52.3	Deep dives (>30 m), faulty restorations (e.g., fillings, crowns), frequent diving, diving experience, older age	Alwohaibi et al., Onose et al., Moyaux et al., Tsur et al., Gougeon et al., Sumen et al.
Periodontal issues	2.8–6.6	Frequent diving, prolonged exposure to pressure changes, older age, longer dive durations (≥300 min)	Onose et al., Shshtari et al., Moyaux et al.

## 4 Discussion

This systematic review synthesized evidence on the prevalence, risk factors, and characteristics of orofacial pain in divers, focusing on barodontalgia, TMJ pain, dental barotrauma, and periodontal issues. The most frequently reported condition was barodontalgia, with prevalence rates ranging from 10.8% to 56.1%, followed by TMJ pain and dental barotrauma. Periodontal issues were less frequent but relevant, with a prevalence of 2.8%−6.6%. These findings confirm the complex interactions between individual and environmental factors in the development of orofacial pain in divers and add new information to the literature. While we observed marked heterogeneity (*I*^2^ > 97%) reflecting diverse study populations and methods, this variability itself confirms that orofacial pain affects divers across all exposure levels. The absence of reporting bias strengthens confidence in these prevalence estimates, despite the need for more standardized future research.

The prevalence of barodontalgia reported in this review corresponds with research conducted by Branco et al. (33) and Zadik and Drucker (4), that underlined atmospheric pressure variations and untreated tooth caries as main causes among divers and aircrews. Their results are supported by this review, which shows that previous dental restorations significantly raise sensitivity to barodontalgia, especially in frequent or deep dives. For military divers, for instance, Alwohaibi et al. (20) found a prevalence of 56.1% with higher pain levels in those who dove 10–50 times year or more. Similarly, Onose et al. (27) determined that barodontalgia had occurred in 17.8% of occupational divers, with frequent diving and old age being established risk factors. These findings point to the importance of offsetting some vulnerabilities, such as poorly sealed fillings and undiagnosed dental pathology, by way of pre-dive dental examinations and improved restorative materials.

This review also highlights significant differences in orofacial pain outcomes among recreational, occupational, and military divers. While Alwohaibi et al. (20) reported elevated barodontalgia (56.1%) and barotrauma (52.3%) in their military cohort, the contrasting 9.3% barotrauma prevalence in Tsur et al.'s study (32) and small number of available military studies (*n* = 2) suggest these estimates may not reflect all military divers. Occupational divers experienced more TMJ pain (15.7%), which was due to repeated jaw tension and constrictive diving equipment (27). Leisure divers, albeit to a lesser extent, were not spared risk, particularly those with existing dental issues or received poor preventive care. As an example, Moyaux et al. noted that barodontalgia happened in 10.8% of civilian divers, wherein age and multiple diving qualifications were overriding risk factors. These differences require individualized prevention in accordance with the unique requirements of each diver group.

TMJ pain was more common in female divers and those with repetitive jaw strain, consistent with findings by Branco et al. (33). This review emphasizes the need for ergonomic interventions, such as better-fitting mouthpieces and equipment adjustments, to mitigate jaw strain, particularly among occupational divers. Frequent dives and longer dive times were found to be significant risk factors for TMJ discomfort, highlighting the need to address behavioral factors, such as jaw clenching, through training and education.

Dental barotrauma, encompassing restoration fractures and prosthesis loss, has a wide prevalence range of 2.3%−52.3%. Zadik and Drucker identified poorly sealed restorations as a primary risk factor, a finding corroborated by the current review. This review adds further granularity by connecting dental barotrauma to specific diving practices, such as deeper dives and repeated exposure to varying atmospheric pressures. Routine pre-dive dental assessments are essential to identify and address vulnerabilities, such as poorly sealed restorations, before they lead to dental injuries.

Although less common (2.8%−6.6%), periodontal problems were ascribed to frequent dives and extended pressure variation. Zadik and Drucker made a brief reference to similar risks; however, they did not elaborate on periodontal pain in divers. The current review presents novel evidence for periodontal pain prevalence and risk factors, including prolonged pressure exposure, inadequate oral hygiene, and existing periodontal disease. These findings support the inclusion of periodontal health in overall preventive planning for divers.

Overall, these findings emphasize the importance of tailored preventive measures to address the unique needs of different diver population. For military divers, enhanced access to dental care along with ergonomic interventions may minimize risks, whereas for leisure divers, it may mean increased awareness of potential dental problems and their management.

### 4.1 Limitations

This review has some limitations, most of which rely on self-reported data, which may reflect certain biases, such as recall bias and subjective variability in reporting pain. The lack of standardized measurement tools and consistent outcome definitions limited the comparability and synthesis of results. Additionally, the predominance of cross-sectional studies means the prevalence rates are influenced by participants' cumulative dive history rather than acute risk per exposure; longitudinal studies measuring incidence per diver-hours would provide more precise risk estimates, though none were available for inclusion. In addition, the absence of data on children and extreme-depth dives (>55 m) reflects gaps in the literature, limiting generalization to these groups. Most of the included studies had cross-sectional designs, limiting the causal effect, and the geographical distribution may only begin to reflect the global nature of diving and risks posed.

### 4.2 Recommendations

The risk of orofacial pain in divers is multicausal. Therefore, education, prevention, and research should be conducted in a multidisciplinary manner to minimize this risk. Regular dental checkups before diving, especially among military and professional divers, are highly feasible and effective, thus providing an immediate benefit in the reduction of the risk of orofacial pain.

Education is a key preventive strategy, which may include predive workshops on jaw relaxation techniques, guidance on properly fitting equipment, and accessible online resources promoting oral health. A multidisciplinary approach involving dentists, diving instructors, and manufacturers is recommended to implement effective preventive measures. For example, ergonomic solutions, such as better mouthpiece designs or equipment adjustments, can help alleviate the most common contributing factors to orofacial discomfort.

Specifically for military and occupational divers, these interventions should be advocated to change policies with the involvement of relevant policymakers and funding bodies, as they are vital for effecting necessary and lasting changes in diving practices and preventive care.

Moreover, the findings have implications for the development of safety protocols and clinical guidelines for practice generally. Is the inclusion of routine dental checkups and education likely to lead to improved health and operational readiness of divers? Thus, guidelines should be preventive, recommending early interventions in conditions with a predisposition to orofacial pain.

Future research should focus on underrepresented groups, such as children and technical divers, while exploring the effects of extreme-depth dives whenever feasible. The standardization of methodologies between studies will provide better comparability and enable meta-analysis. Closing these gaps in knowledge will provide the field with more tailored and effective preventative measures to ensure diver safety and wellbeing.

## Data Availability

The original contributions presented in the study are included in the article/supplementary material, further inquiries can be directed to the corresponding author.
